# Supplementary material for: A tRNA modification balances carbon and nitrogen metabolism by regulating phosphate homeostasis
Source: eLife. 2019 Jul 1;8:e44795. doi: 10.7554/eLife.44795 (PMC6688859; doi:10.7554/eLife.44795)
Supplement: Supplementary file 1. [file elife-44795-supp1.docx]

**Gupta et al**

**Supplementary File 1**

**Strains used in this study**

| **Strain** | **Genotype** | **Source** |
| --- | --- | --- |
| CEN.PK | *Mat a* | van Dijken et al, Enzyme Microb Technol, 2000 |
| *uba4*Δ | *Mat a uba4*Δ::*NAT* | This study |
| *ncs2*Δ | *Mat a ncs2*Δ::*NAT* | This study |
| *GCN4-HA SUI2-Flag* | *Mat a GCN4-HA-KanMX SUI2-Flag-NAT* |  |
| *uba4*Δ *GCN4-HA SUI2-Flag* | *Mat a uba4*Δ*::Hyg GCN4-HA-KanMX SUI2-Flag-NAT* | This study |
| *ncs2*Δ *GCN4-HA SUI2-Flag* | *Mat a ncs2*Δ*::Hyg GCN4-HA-KanMX SUI2-Flag-NAT* | This study |
| *ncs6*Δ *GCN4-HA SUI2-Flag* | *Mat a ncs6*Δ::*Hyg* *GCN4-HA-KanMX SUI2-Flag-NAT* | This study |
| *gcn2*Δ | *Mat a gcn2*Δ::*Hyg* | This study |
| *uba4*Δ *gcn2*Δ | *Mat a uba4*Δ::*NAT gcn2*Δ::*Hyg* | This study |
| *ncs2*Δ *gcn2*Δ | *Mat a ncs2*Δ::*NAT gcn2*Δ::*Hyg* | This study |
| *tps2*Δ | *Mat a tps2*Δ::*Hyg* | This study |
| *uba4*Δ *tps2*Δ | *Mat a uba4*Δ::*NAT tps2*Δ::*Hyg* | This study |
| *ncs2*Δ *tps2*Δ | *Mat a ncs2*Δ::*NAT tps2*Δ::*Hyg* | This study |
| *bar1*Δ | *Mat a bar1*Δ::*Hyg* | This study |
| *uba4*Δ *bar1*Δ | *Mat a uba4*Δ::*NAT bar1*Δ::*Hyg* | This study |
| *ncs2*Δ *bar1*Δ | *Mat a ncs2*Δ::*NAT bar1*Δ::*Hyg* | This study |
| *uba4C225A* | *Mat a uba4C225A::KanMX* | Laxman et al, Cell, 2013 |
| *uba4C397A* | *Mat a uba4C397A::KanMX* | Laxman et al, Cell, 2013 |
| *PHO12-Flag* | *Mat a PHO12-Flag-Hyg* | This study |
| *uba4*Δ *PHO12-Flag* | *Mat a uba4*Δ::*NAT PHO12-Flag-Hyg* | This study |
| *ncs2*Δ *PHO12-Flag* | *Mat a ncs2*Δ::*NAT PHO12-Flag-Hyg* | This study |
| *PHO84-Flag* | *Mat a PHO84-Flag-Hyg* | This study |
| *uba4*Δ *PHO84-Flag* | *Mat a uba4*Δ::*NAT PHO84-Flag-Hyg* | This study |
| *ncs2*Δ *PHO84-Flag* | *Mat a ncs2*Δ::*NAT PHO84-Flag-Hyg* | This study |
| *pho4*Δ | *Mat a pho4*Δ::*Hyg* | This study |
| *gcn4*Δ | *Mat a gcn4*Δ::*Hyg* | This study |
| *uba4*Δ *gcn4*Δ | *Mat a uba4*Δ::*NAT gcn4*Δ*::Hyg* | This study |
| *pho85*Δ *GCN4-HA* | *Mat a pho85*Δ::*Hyg GCN4-HA-KanMX* | This study |
| *pho80*Δ | *Mat a pho80*Δ::*Hyg* | This study |
| *uba4*Δ *pho80*Δ | *Mat a uba4*Δ::*NAT pho80*Δ*::Hyg* | This study |
| *ncs2*Δ *pho80*Δ | *Mat a ncs2*Δ::*NAT pho80*Δ*::Hyg* | This study |
