# Supplementary material for: A tRNA modification balances carbon and nitrogen metabolism by regulating phosphate homeostasis
Source: eLife. 2019 Jul 1;8:e44795. doi: 10.7554/eLife.44795 (PMC6688859; doi:10.7554/eLife.44795)
Supplement: Supplementary file 2. [file elife-44795-supp2.docx]

**Gupta et al**

**Supplementary File 2**

**Plasmids used in this study**

| **Plasmid** | **Description** | **Source** |
| --- | --- | --- |
| pGL3-Basic | Luciferase reporter vector; firefly luciferase cDNA (AmpR) | Addgene |
| pSL80 | p417-TEF1 plasmid (*CEN*, KanR) | (Wu and Tu, MBC, 2011) |
| SL147 | Luciferase cDNA from pGL3-Basic in pSL80 | This study |
| SL148 | WT Gcn4 uORF (500 bp region upstream of Gcn4 ATG start codon) fused to codons encoding first 55 amino acids of Gcn4 in SL147 (*CEN*, KanR) | This study |
| SL149 | Mutated Gcn4 uORF1 (uORF1* denotes point mutation ATG→AGG at uORF1 translation start site, in 500 bp region upstream of Gcn4 ATG start codon) fused to codons encoding first 55 amino acids of Gcn4 in SL147 (*CEN*, KanR) | This study |
| SL150 | Mutated Gcn4 uORF4 (uORF4* denotes point mutation ATG→AGG at uORF4 translation start site, in 500 bp region upstream of Gcn4 ATG start codon) fused to codons encoding first 55 amino acids of Gcn4 in SL147 (*CEN*, KanR) | This study |
