# Supplementary material for: A tRNA modification balances carbon and nitrogen metabolism by regulating phosphate homeostasis
Source: eLife. 2019 Jul 1;8:e44795. doi: 10.7554/eLife.44795 (PMC6688859; doi:10.7554/eLife.44795)
Supplement: Supplementary file 3. [file elife-44795-supp3.docx]

**Gupta et al**

**Supplementary File 3**

**Mass transitions for detection of metabolites**

| **Metabolite** | **Q1/Q3 (Parent/Product)** |  |
| --- | --- | --- |
| **Amino acids** |  |  |
| Arginine | 175.2/60.0 |  |
| Lysine | 147.0/84.1 |  |
| Proline | 116.0/70.0 |  |
| Aspartate | 134.1/74.0 |  |
| Threonine | 120.0/56.0 |  |
| Asparagine | 133.1/74.0 |  |
| Glutamine | 147.0/84.1 |  |
| Glutamate | 148.1/84.1 |  |
| Cysteine | 122.2/59.0 |  |
| Methionine | 150.1/56.0 |  |
| Histidine | 156.0/110.0 |  |
| Alanine | 90.0/44.0 |  |
| Serine | 106.0/60.0 |  |
| Isoleucine | 132.0/86.0 |  |
| Valine | 118.0/72.0 |  |
| Leucine | 132.0/86.0 |  |
| Phenylalanine | 166.0/120.0 |  |
| Tryptophan | 205.0/146.0 |  |
| Tyrosine | 182.2/136.0 |  |
| **Nucleotides** |  |  |
| Uridine 5'-monophosphate (UMP) | 325/113 |  |
| Adenosine 5’-monophosphate (AMP) | 348/136 |  |
| Guanosine 5’-monophosphate (GMP) | 364/152 |  |
| Cytidine 5’-monophosphate (CMP) | 324/112 |  |
| **^15^N-labelling experiments** |  |  |
| Adenosine 5’-monophosphate (AMP) | 348/136 | Product has all nitrogens |
| ^15^N-AMP_1 | 349/137 |  |
| ^15^N-AMP_2 | 350/138 |  |
| ^15^N-AMP_3 | 351/139 |  |
| ^15^N-AMP_4 | 352/140 |  |
| ^15^N-AMP_5 | 353/141 |  |
| Guanosine 5’-monophosphate (GMP) | 364/152 |  |
| ^15^N-GMP_1 | 365/153 |  |
| ^15^N-GMP_2 | 366/154 |  |
| ^15^N-GMP_3 | 367/155 |  |
| ^15^N-GMP_4 | 368/156 |  |
| ^15^N-GMP_5 | 369/157 |  |
| Cytidine 5’-monophosphate (CMP) | 324/112 |  |
| ^15^N-CMP_1 | 325/113 |  |
| ^15^N-CMP_2 | 326/114 |  |
| ^15^N-CMP_3 | 327/115 |  |
| Aspartate | 134.1/74.0 | Product has a nitrogen |
| ^15^N-Aspartate_1 | 135.1/75.0 |  |
| Glutamine | 147.0/84.1 | Product has only one nitrogen |
| ^15^N-Glutamine_1 | 148.0/85.1 |  |
| ^15^N-Glutamine_2 | 149.0/85.1 |  |
|  |  |  |
| **^13^C-labelling experiments** |  |  |
| Adenosine 5’-monophosphate (AMP) | 348/136 | Product has 5 carbons coming from the nitrogen base. The rest of the carbons form the ribose ring. |
| ^13^C-AMP_5 (labelled ribose) | 353/136 |  |
| ^13^C-AMP_6 (labelled ribose) | 354/137 |  |
| ^13^C-AMP_7 (labelled ribose) | 355/138 |  |
| ^13^C-AMP_8 (labelled ribose) | 356/139 |  |
| ^13^C-AMP_9 (labelled ribose) | 357/140 |  |
| ^13^C-AMP_10 (labelled ribose) | 358/141 |  |
| Adenosine 5’-diphosphate (ADP) | 428/136 |  |
| ^13^C-ADP_5 (labelled ribose) | 433/136 |  |
| ^13^C-ADP_6 (labelled ribose) | 434/137 |  |
| ^13^C-ADP_7 (labelled ribose) | 435/138 |  |
| ^13^C-ADP_8 (labelled ribose) | 436/139 |  |
| ^13^C-ADP_9 (labelled ribose) | 437/140 |  |
| ^13^C-ADP_10 (labelled ribose) | 438/141 |  |
| Adenosine 5’-triphosphate (ATP) | 508/136 |  |
| ^13^C-ATP_5 (labelled ribose) | 513/136 |  |
| ^13^C-ATP_6 (labelled ribose) | 514/137 |  |
| ^13^C-ATP_7 (labelled ribose) | 515/138 |  |
| ^13^C-ATP_8 (labelled ribose) | 516/139 |  |
| ^13^C-ATP_9 (labelled ribose) | 517/140 |  |
| ^13^C-ATP_10 (labelled ribose) | 518/141 |  |
| Guanosine 5’-monophosphate (GMP) | 364/152 |  |
| ^13^C-GMP_5 (labelled ribose) | 369/152 |  |
| ^13^C-GMP_6 (labelled ribose) | 370/153 |  |
| ^13^C-GMP_7 (labelled ribose) | 371/154 |  |
| ^13^C-GMP_8 (labelled ribose) | 372/155 |  |
| ^13^C-GMP_9 (labelled ribose) | 373/156 |  |
| ^13^C-GMP_10 (labelled ribose) | 374/157 |  |
| Trehalose | 341.3/179.3 | Product has 6 carbons of which either 3 or 6 could be labelled |
| ^13^C-Trehalose_6 | 347.3/185.3 |  |
| ^13^C-Trehalose_12 | 353.3/185.3 |  |
| 2/3-Phosphoglycerate (2/3PG) | 185/97 | Monitoring the phosphate release |
| ^13^C-2/3PG_3 | 188/97 |  |
| 6-Phosphogluconate (6PG) | 259/97 |  |
| ^13^C-6PG_6 | 281/97 |  |
| 5-Ribose/ribulose-5-phosphate (R5P) | 229/97 |  |
| ^13^C-R5P_5 | 234/97 |  |
| Sedoheptulose-7-phosphate (S7P) | 289/97 |  |
| ^13^C-S7P_2 | 291/97 |  |
| ^13^C-S7P_5 | 294/97 |  |
| ^13^C-S7P_7 | 296/97 |  |
